# Evaluation of acne vulgaris patient care in outpatient clinics: guideline adherence and patient education

**DOI:** 10.55730/1300-0144.6355

**Published:** 2026-05-20

**Authors:** Mefküre DURMUŞ, Betül Sena ÇAVDAR, İbrahim Etem ARICA, Sena USTAÖMER, Sena GÜZEL KARAHAN, Seçil ÇOLAK, Selcen ŞENOL

**Affiliations:** 1 Department of Clinical Pharmacy, Faculty of Pharmacy, Karadeniz Technical University, Trabzon, Turkiye; 2 Faculty of Pharmacy, Karadeniz Technical University, Trabzon, Turkiye; 3 Department of Dermatology and Venerology, Faculty of Medicine, Karadeniz Technical University Trabzon, Turkiye; 4 Department of Clinical Pharmacy, Faculty of Pharmacy, İnönü University, Malatya, Turkiye; 5 Department of Physical Medicine and Rehabilitation, Garnet Health Medical Center, New York, NY, USA

**Keywords:** Antiacne medication, guideline adherence, patient education, acne vulgaris, isotretinoin

## Abstract

**Background/aim:**

Acne vulgaris, affecting over 85% of adolescents, is the most prevalent chronic inflammatory skin disorder. Its management is severity-based, with specific drug selections and combinations according to guidelines. However, adherence to these guidelines and the adequacy of patient counseling remain uncertain in real-world practice. The aim in this study was to evaluate prescribing patterns in acne management, assess compliance with guidelines, and promote awareness among healthcare professionals.

**Materials and methods:**

A prospective observational study was conducted in the dermatology outpatient clinic of a tertiary hospital in Türkiye between February and May 2025. Demographic and clinical data were collected, and acne severity was graded according to the Türkiye Dermatology Association (TDA) criteria. Prescriptions were evaluated for guideline concordance with American Dermatology Association (ADA) 2016/2024, National Institute for Health and Care Excellence (NICE) 2021, and TDA 2018. Potential drug–drug interactions and the adequacy of clinical teaching for acne medication were also assessed through a multidisciplinary evaluation. Statistical analyses were performed using descriptive methods, the Kruskal–Wallis test, Pearson’s chi-squared test, and Fisher’s exact test, as appropriate.

**Results:**

Oral isotretinoin, topical retinoids, and topical antibiotics were the most frequently prescribed agents. While isotretinoin use was largely appropriate, deviations from guideline recommendations, particularly oral antibiotic monotherapy, were observed. Some drug–drug interactions and limitations in structured patient counseling were also identified. Among the clinical guidelines, adherence was highest to the ADA guidelines, followed by the TDA guidelines. Moreover, the severity of the disease appeared to influence which guideline was followed.

**Conclusion:**

Although treatment largely followed severity-based recommendations, gaps in guideline adherence and patient education highlight the need for improved prescribing practices and interprofessional collaboration in acne management.

## 1. Introduction

Acne vulgaris is the most common chronic inflammatory dermatological disease, affecting more than 85% of adolescents [1,2]. The management of acne vulgaris depends on disease severity. Mild cases are generally treated with benzoyl peroxide (BPO) or topical retinoids, alone or in combination with topical antibiotics. In moderate acne, combination regimens with topical or oral antibiotics and retinoids are preferred, while hormonal agents may be considered in females. In severe disease, oral antibiotics with topical agents are commonly used, and oral isotretinoin may be initiated as first-line therapy [3]. Evidence from 29 randomized clinical trials supports this severity-based approach, showing the efficacy of topical antibiotic–retinoid combinations in mild to moderate cases, oral antibiotics in moderate to severe disease, and isotretinoin in severe or refractory nodular acne [4].

Guidelines from major authorities, including the American Dermatology Association (ADA), National Institute of Health and Clinical Excellence (NICE), and Türkiye Dermatology Association (TDA), provide recommendations on treatment selection, drug combinations, and severity-based use in acne management. While appropriate treatment selection and its duration are critical for effective outcomes, the extent to which these guidelines are followed in clinical practice and the presence of variations in prescribing patterns remain uncertain, particularly in tertiary healthcare settings, where complex or resistant cases are more frequently encountered.

Prescription pattern studies highlight the frequent use of benzoyl peroxide, topical antibiotics, and oral doxycycline in acne treatment, yet evidence regarding guideline adherence remains limited [5].

Appropriate use of medications in acne vulgaris is a key determinant of treatment success. Providing patients with effective information and warnings during therapy enhances adherence, reduces adverse effects, and improves overall satisfaction [6,7]. However, the literature remains limited on how well prescribed medications align with treatment guidelines and on the adequacy of patient counseling. To address these gaps, in the present study we evaluated physicians’ drug choices in the management of acne vulgaris, assessed their compliance with current guidelines, and aimed to raise awareness among healthcare professionals—such as physicians and pharmacists—regarding treatments requiring caution and their consistency with evidence-based recommendations.

## 2. Materials and methods

This research was a prospective observational study performed over a 3-month period in the dermatology outpatient clinic of a tertiary care hospital in Türkiye. The admissions between 06.02.2025 and 06.05.2025 were evaluated. The inclusion criteria were as follows: having acne vulgaris, agreeing to participate in the study, and signing the consent form. The exclusion criteria were as follows: declining to participate in the study and having drug eruptions. The study was approved by the Scientific Research Ethics Committee of Karadeniz Technical University’s Faculty of Medicine with the protocol number 2024/224, dated 5 February, 2025.

Demographic and clinical data were recorded. At the end of the study period, a meeting with two dermatologists and three pharmacists was held to evaluate the guideline concordance and clinical teaching for acne medication therapy. The issues unveiled were as follows: acne prescription patterns of dermatologists, guidelines-concordant treatment, possible drug–drug interactions in the acne vulgaris patient population, and appropriateness of clinical teaching. The findings from the meeting were recorded.

The grading of acne vulgaris was determined in accordance with the TDA and was based on the current clinical findings at the outpatient visit during the study period rather than the severity at treatment initiation. However, for the evaluation of guideline adherence, the acne grade and prescribed treatment at the initial presentation were taken into account. The ADA 2016 and 2024 guidelines were presumed to be the same and defined as the ADA guidelines except for oral retinoid therapy (isotretinoin), which differed between them. Patients with psychosocial load were identified as candidates for oral isotretinoin regardless of the disease severity for the ADA 2024 guidelines.

The prescription patterns were evaluated and defined as guideline concordant or not. The guidelines evaluated were the ADA 2016 and 2024, NICE 2021, and TDA 2018.

### 2.1. Definitions based on the criteria according to the guidelines

#### ADA guidelines

Patients with mild grade were categorized as guideline concordant if they received the following three patterns: BPO or topical retinoid alone, BPO + topical antibiotic, or BPO + topical retinoid + topical antibiotic. Patients with moderate grade were categorized as guideline concordant if they received the following three patterns: BPO + topical antibiotic, BPO + topical retinoid, or oral antibiotic + topical retinoid + BPO + topical antibiotic. Patients with severe grade were categorized as guideline concordant if they received the following four patterns: oral isotretinoin, oral antibiotic + BPO + topical antibiotic, oral antibiotic + topical retinoid + BPO, or oral antibiotic + topical retinoid + BPO + topical antibiotic [8,9]. Those criteria were feasible for the ADA 2016 and ADA 2024 guidelines; however, oral isotretinoin was considered as ADA 2024 guideline concordant for three grades [9].

#### NICE 2021 guidelines

Patients with any grade were categorized as guideline concordant if they received the following two patterns: topical adapalene + topical BPO or topical tretinoin + topical clindamycin. Patients with mild/moderate grade were categorized as guideline concordant if they received the following pattern: topical BPO + topical clindamycin. Patients with moderate/severe grade were categorized as guideline concordant if they received the following three patterns: topical adapalene + topical BPO + oral antibiotic, topical azelaic acid + oral antibiotic, or oral isotretinoin [10].

#### TDA 2018 guidelines

Patients with mild grade were categorized as guideline concordant if they received the following two patterns: topical retinoid or topical retinoid + azelaic acid. Patients with moderate grade were categorized as guideline concordant if they received the following four patterns: topical retinoid + topical antibiotic, topical retinoid + topical antibiotic + azelaic acid, oral antibiotic + BPO or oral antibiotic + topical retinoid, or oral antibiotic + topical retinoid ± BPO/azelaic acid. Patients with severe grade were categorized as guideline concordant if they received the following three patterns: oral antibiotic + topical retinoid + BPO, oral isotretinoin, or oral antibiotic + topical retinoid ± BPO/azelaic acid [11].

In the present study, the following agents were included in the prescribed antiacne medications: adapalene and retinoic acid as topical retinoids, doxycycline as an oral antibiotic, erythromycin and clindamycin as topical antibiotics, isotretinoin as oral retinoid, BPO, and azelaic acid. No other drugs were prescribed in the study.

### 2.2. Sample size

The required sample size was determined based on available evidence from the acne vulgaris prescribing-pattern literature. Therefore, a cross-sectional observational study analyzing 135 prescriptions in patients with acne vulgaris was used as the most relevant methodological reference [1]. In addition, all patients who met the inclusion criteria during the 3-month study period were consecutively included in the study. Accordingly, the final sample size was considered adequate and comparable for evaluating prescribing patterns and guideline concordance in patients with acne vulgaris.

### 2.3. Statistical analysis

The statistical analyses were performed using IBM SPSS Statistics version 23. Continuous variables were tested for normality and expressed as median and interquartile range (IQR), and comparisons among acne severity groups were performed using the Kruskal–Wallis test. Categorical variables were presented as numbers and percentages. Pearson’s chi-squared test was used for categorical comparisons when expected cell counts were adequate, whereas Fisher’s exact test was applied when expected frequencies were small or zero. When overall categorical comparisons were statistically significant, post hoc pairwise comparisons of column proportions with Bonferroni correction were performed. A two-sided p-value <0.05 was considered statistically significant.

## 3. Results

### 3.1. Demographic and clinical data

A total of 145 patients were evaluated prospectively over 3 months (06/02/2025–06/05/2025). Males comprised 21.3% (n = 31) of the patients. The median age was 19 years (IQR: 18–21.5). The median age of the females and males was 20 (IQR: 18–22) and 18 (IQR: 17–20), respectively. The grades of acne vulgaris were 31% mild, 47% moderate, and 22% severe. Males were significantly more frequent in the severe category. The relationship between demographics and grades is given in Table 1. Age and sex were not correlated with the acne prescription pattern, but oral antibiotic (doxycycline) was associated with the male sex. The demographic data and prescription patterns are given in Table 2. Age and guideline-concordance were not correlated. Fourteen patients (9.7%) had at least one comorbidity and the most common comorbidity was anxiety (3.4%), followed by asthma (2.1%) and depression (1.4%). Polypharmacy was observed in only eight patients, while drug–drug interactions were identified in six patients: three classified as category D, two as category C, and one as category B. The category D interactions included ferrous sulfate–doxycycline and clindamycin/BPO–erythromycin/tretinoin combinations, which should be advised to be administered at different times of the day.

### 3.2. The prescription patterns of antiacne medication

**Figure 1:**
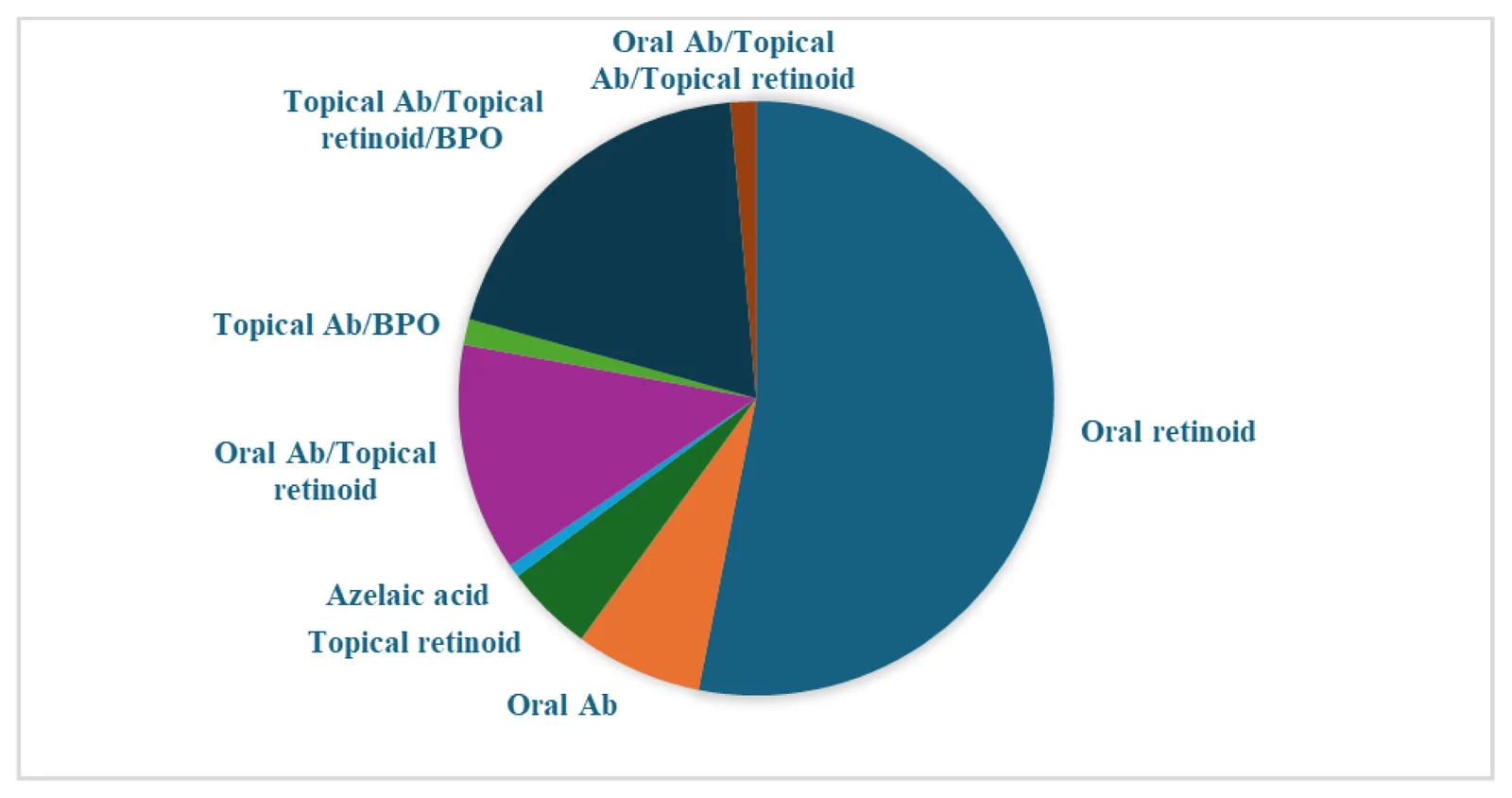
Distribution of prescribed antiacne medications. Ab: antibiotic.

The most common prescribed antiacne medication was oral isotretinoin (53.1%). Topical retinoids (38.62%), topical antibiotics (22%), and oral antibiotics (20.7%) followed oral isotretinoin regarding frequency. Topical azelaic acid was detected in one patient. The distribution of the prescribed antiacne medication is shown in Figure 1. The total number of antiacne medications per patient was 1.09. Oral antibiotic (doxycycline) had significantly higher rates in the patients with moderate or severe grades. Additionally, topical retinoids, topical antibiotics, and BPO were the medications frequently prescribed to patients with mild disease (Table 1).

### 3.3. Guideline concordance

Oral antibiotic concordance with the ADA and NICE guidelines was significantly higher in the moderate cases, although no difference was found between the TDA guidelines and cases of any grade. Oral retinoid concordance was significantly higher in the severe cases for the three guidelines. Topical antibiotic concordance was correlated with the ADA guidelines in mild and moderate cases, although there was no relationship with the TDA or NICE guidelines. Topical retinoid concordance with the ADA and NICE guidelines was significantly associated with the mild or moderate cases. BPO/clindamycin concordance with the ADA guidelines was significantly associated with the mild or moderate cases, although no relationship was found between the other guidelines and cases of any grade.

The overall percentages of concordance with the guidelines were as follows: 84.1% (122 patients) for ADA 2024, 62.1% (90 patients) for ADA 2016, 46.9% (68 patients) for TDA, and 40% (58 patients) for NICE. In mild cases, concordance with the ADA guidelines was significantly higher, whereas, in severe cases, concordance with the NICE and TDA guidelines was significantly higher (Table 1).

### 3.4. Mono-, dual-, and triple-therapy patterns and guideline concordance

**Figure 2:**
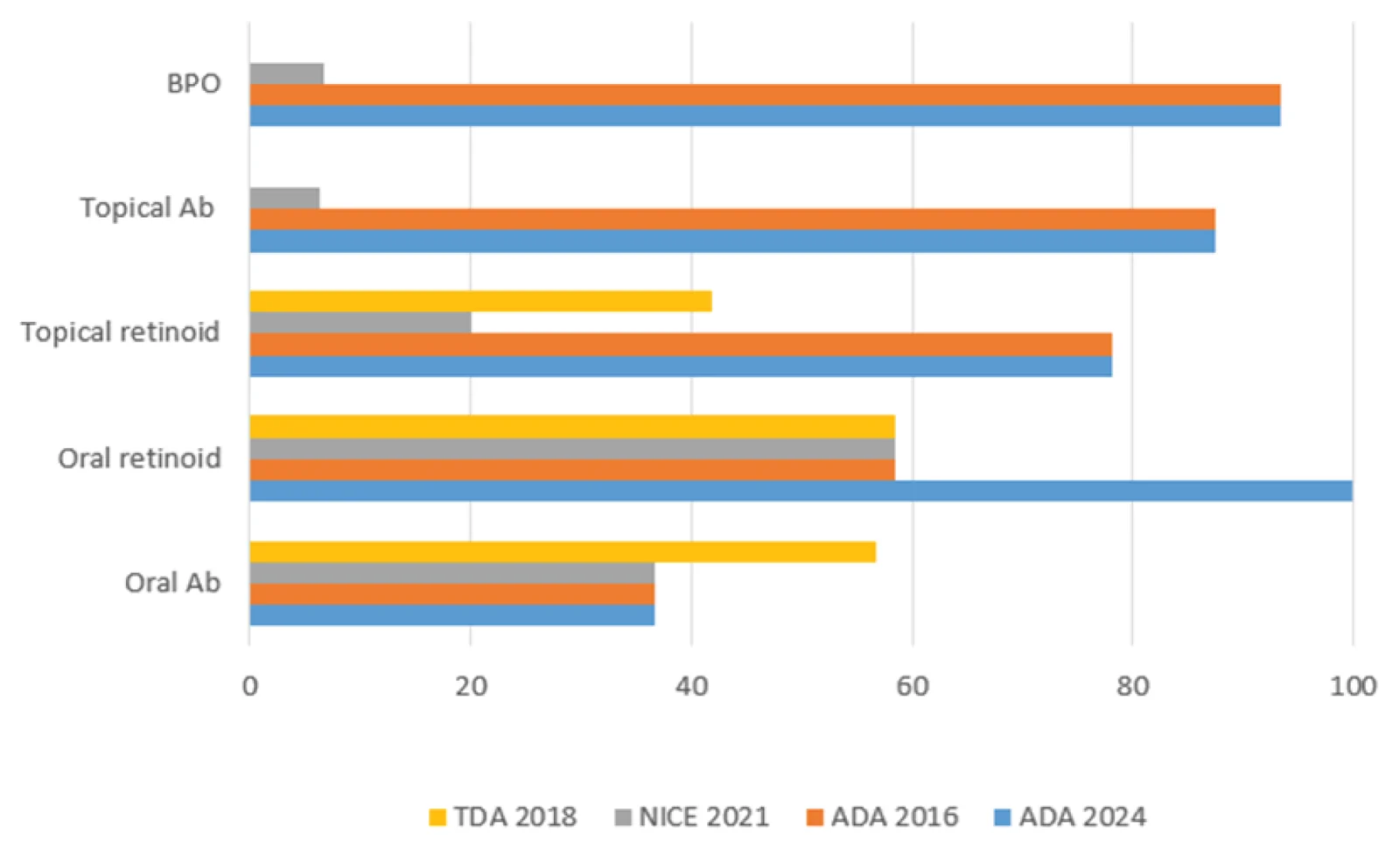
Comparison of guideline-concordant percentages of prescribed antiacne medications. Ab: antibiotic.

Monotherapy with oral retinoids increased concordance with NICE and TDA, whereas monotherapy with oral antibiotics decreased concordance. Dual therapy with an oral antibiotic plus a topical retinoid was significantly concordant with TDA. In contrast, triple therapy with a topical retinoid, topical antibiotic, and benzoyl peroxide was concordant only with ADA, but significantly discordant with all other guidelines. Therapy patterns and their concordance with the guidelines are presented in Table 3. Oral antibiotics were the most guideline-concordant medication with TDA, while oral retinoids showed the highest concordance with ADA 2024, and topical retinoids and benzoyl peroxide with ADA. Descriptive data regarding therapy patterns and concordance are presented in Figure 2.

### 3.5. Clinical teaching about antiacne medication by dermatologists

Dermatologists’ success rates in clinical teaching of antiacne medications were above 50%. Topical retinoids (75%) and oral retinoids (71.4%) had higher clinical teaching scores compared to other therapies. The least frequently provided counseling points for retinoids were skin color changes with topical tretinoin (0%), avoidance of blood donation (5.2%), and caution with vitamin A–A-containing supplements or medications during oral retinoid therapy (2.6%). For nonretinoid agents, the least emphasized advice was the application of benzoyl peroxide to the entire affected area (3.3%) and the timing of doxycycline administration in cases of specific drug–drug interactions (ferrous sulfate–doxycycline), which was noted in only 10% of cases. Counseling points for antiacne medications and their respective frequencies are summarized in Table 4.

## 4. Discussion

In the present study, the characteristics of patients with acne vulgaris and the management practices of dermatologists in Türkiye were evaluated. A comparative evaluation of three different guidelines (United States, United Kingdom, and Türkiye) is provided and the differences among them are evaluated. Despite its clinical importance, the teaching of antiacne medications has been largely overlooked by both pharmacists and dermatologists. Our findings draw attention to this neglected aspect and emphasize the need to strengthen awareness and education regarding the clinical use of topical and systemic antiacne therapies.

The demographic characteristics of acne vulgaris patients may vary across different countries. In our study conducted in Türkiye, the median age of the patients was 19 years, which is consistent with other studies reporting median ages ranging from 17.8 to 23.7 years [12,13]. In a Colombian study of 21,604 acne vulgaris patients, a median age of 20.8 years with 60.7% females was reported [12]. In our study, the median age was similar, although the proportion of women was higher (78.6%), possibly due to geographic or ethnic differences.

Dunaway and Fleischer analyzed 921 acne vulgaris patients over 7 years, reporting 61.9% female and 86.4% white patients [13]. The higher female proportion in our study (78.6%) may have been related to the inclusion of only white patients, which is consistent with literature reports ranging from 65.4% to 80% [12]. Several drugs, including corticosteroids, androgens, chloroquine, isoniazid, and lithium, are known to exacerbate acne lesions. Others, such as cyclosporine, vitamin B12, barbiturates, quinidine, tricyclic antidepressants, and tacrolimus, have been implicated with limited evidence, while agents like vitamins B1 and B6, propylthiouracil, voriconazole, rifampicin, and ethambutol are only occasionally associated with acne vulgaris [14,15]. Valladale et al. reported that corticosteroids and androgens were the most frequently used acne-related drugs (17.2%) [12]. In our study, tacrolimus (0.7%) and hydroxychloroquine (0.7%) were rarely used and no association with acne was observed.

In a study from Colombia, acne vulgaris management was analyzed and doxycycline was the most frequently prescribed oral antibiotic (72.1%), similar to our study, where it was the only oral antibiotic prescribed. In that study, oral isotretinoin and topical retinoids were prescribed in 7.6% and 49.3% of cases, respectively, while monotherapy, dual therapy, and triple therapy were observed in 59.6%, 35.7%, and 4.7% of patients [12]. In contrast, the prescription patterns in our study differed, with oral doxycycline, oral isotretinoin, and topical retinoids accounting for 20.7%, 53.1%, and 38.6%, respectively, and monotherapy, dual therapy, and triple therapy rates of 64.6%, 13.6%, and 20.5%. These discrepancies likely reflect differences in prescribing practices influenced by regional and geographic factors. In previous studies, it was reported that topical agents are preferred in mild acne and systemic agents in severe forms [12,16]. Similarly, we observed higher use of topical retinoids, antibiotics, and benzoyl peroxide in mild cases and systemic antibiotics in severe cases.

Inappropriate oral antibiotic monotherapy is a common concern in acne management [12]. Although this was observed in only 6.8% of our patients, it still reflects guideline-discordant prescribing. In our study, the combination of oral antibiotics with topical retinoids was significantly concordant with the TDA guideline, whereas concordance with the other guidelines did not reach statistical significance.

Fleischer demonstrated that dermatologists’ prescriptions for atopic dermatitis were often not guideline concordant and highlighted the benefit of reviewing clinical guidelines [17]. Our results are consistent with this observation, underscoring the importance of guideline adherence in clinical practice. Dunaway and Fleischer reported topical retinoids as the most common treatment (46.7%), followed by oral tetracyclines (35.3%) and topical antibiotic/BPO (25%). Oral isotretinoin use was lower (19.9%) and antibiotic monotherapy accounted for 14.8% [13]. In our study, topical retinoids, oral tetracyclines, benzoyl peroxide, and topical antibiotics were prescribed at rates comparable to those reported by Dunaway and Fleischer, although oral isotretinoin was used far more frequently in our cohort (53.1%). This relatively high rate should be interpreted with caution. Patients with treatment-resistant acne, previous treatment failure, and greater psychosocial burden may have been more frequently encountered because our study was conducted in a tertiary-care center. In addition, acne severity reported in the descriptive analyses was classified according to clinical findings at the outpatient visit rather than at treatment initiation. Therefore, some patients categorized as having mild acne may have initially presented with more severe disease and improved under ongoing isotretinoin therapy. Accordingly, this finding does not necessarily indicate inappropriate prescribing, although potential overuse cannot be completely excluded. Regarding antibiotic prescribing patterns, while their study underscored inappropriate oral antibiotic monotherapy, we likewise identified oral doxycycline monotherapy in 6.8% of cases, a practice significantly discordant with guideline recommendations (p < 0.05).

In mild to moderate acne, topical therapy was the primary choice, whereas systemic therapy was preferred as first-line treatment in moderate to severe cases. In that study, combinations of oral antibiotics with topical retinoids and topical antibiotics with benzoyl peroxide or topical retinoids improved patient outcomes. Additionally, topical antibiotic monotherapy was considered inappropriate and should be avoided [18]. In our study, topical therapy was primarily preferred in mild cases, whereas systemic therapy was the main choice in severe cases. In mild cases, topical retinoid monotherapy was prescribed in 4.8% of patients. Topical retinoids were more frequently used in combination regimens, either with oral antibiotics in dual therapy (12.4%) or with topical antibiotics and benzoyl peroxide in triple therapy (19.3%). These findings indicate that topical retinoids were commonly prescribed in accordance with guideline recommendations in our study. Additionally, in our study, topical antibiotics were prescribed appropriately. Topical retinoid–BPO–topical antibiotic triple therapy was prescribed in significant accordance with the ADA guidelines but was discordant with the TDA and NICE guidelines. In this regard, we thought that the ADA guidelines differed from the others. Additionally, in our study, clinicians in Türkiye predominantly adhered to ADA 2024 (84.1%) or ADA 2016 (62.1%) guidelines. Topical retinoids were associated with adverse effects on the skin (dryness, thinning, and redness), requiring gradual dose escalation and the use of sunscreen and moisturizers. BPO was noted to have a bleaching effect on hair and clothes [18]. Warnings about these issues should be shared with patients. In our study, 75% and 62.5% of such warnings were provided by clinicians; these rates should be increased to 100% by both clinicians and pharmacists. In another study, it was noted that although guidelines included recommendations to improve the effectiveness of topical therapy and reduce adverse effects, inconsistencies were present between them [19]. We reviewed the ADA, TDA, and NICE guidelines with respect to warnings related to antiacne medications. Warnings such as “skin color changes with topical retinoids, avoiding blood donation and caution eating food or supplements containing vitamin A with oral retinoid, and severe diarrhea and potential drug–drug interactions with oral doxycycline” were generally overlooked by clinicians. Therefore, these issues should be emphasized among healthcare providers. In one study, the usefulness of NICE guidelines was reported to be 69% [19]. In our study, adherence to the NICE guidelines was 40%, with oral retinoids being the most guideline-concordant medication, at 58%. In a tertiary hospital study from India, acne vulgaris cases were distributed as mild (23.7%), moderate (41.5%), and severe (29.6%), whereas in our study, the rates were 31%, 46%, and 22.1%, with mild cases being the least and severe cases the most frequent [1]. In a study evaluating 107 prescriptions of moderate-to-severe acne cases for their appropriateness according to the NICE guidelines, only 9.8% were found to be guideline concordant. In contrast, the concordance rate in our study was higher, at 40% [20]. In Indonesia, the effectiveness of standard acne vulgaris treatment was investigated in a retrospective study including 2 years of data from 131 patients. Similar to our study, the majority of cases were in women and patients with moderate disease, and the median age of females (23 years) was higher than that of males (19 years) [21].

The present study has several limitations. First, it was a single-center observational study, which may limit the generalizability of the findings to other healthcare settings. Second, prescribing practices were evaluated under routine clinical conditions and no educational intervention was implemented before reassessing guideline adherence. Future multicenter studies incorporating physician education and repeated postintervention evaluations may provide more robust evidence regarding improvement in guideline-concordant prescribing practices. Another limitation is that current acne severity at the outpatient visit may not always reflect baseline severity at treatment initiation. However, guideline adherence was assessed according to the initial presentation records. The main strengths of the study are its prospective design, the assessment of how patient education was provided in routine practice, and the evaluation of prescribing patterns according to three evidence-based sets of guidelines.

In conclusion, we think that, in Türkiye, the high patient load within the healthcare system limits physicians’ ability to provide comprehensive care in the time required for each patient. We evaluated both the communication of antiacne medication warnings to patients and the relationship between guideline concordance and acne severity. Our findings indicate that clinicians were more guideline concordant in severe cases. Overall, greater attention should be given to patient education in acne treatment, and clinical pharmacists should be integrated into dermatology clinics to support both education and guideline adherence.

## Figures and Tables

**Table 1:** Association of acne vulgaris severity with demographic, clinical, prescription characteristics, and guideline concordance.

Variables	Acne vulgaris disease severity grades n (%)			Significance

Demographic characteristics, n (%)	Mild, 45 (31)	Moderate, 68 (46.9)	Severe, 32 (22.1)	p

**Age, median (IQR)**	20 (18.5–21)	19.5 (17–22)	19 (17.3–22)	0.843*

**Sex** **	a	a,b	b	***0.038*** ^
Female, 114 (78.6)	41 (91.1)	51 (75)	22 (68.8)	
Male, 31 (21.3)	4 (8.9)	17 (25)	10 (31.3)	

**Smoker**, 12 (8.3)	5 (11.1)	5 (7.4)	2 (6.3)	0.732+

**Alcohol use**, 2 (1.4)	0	1 (1.5)	1 (3.1)	0.493+

**Educational status**				
Primary school, 2 (1.4)	0	1 (1.5)	1 (3.1)	
High school, 49 (33.8)	13 (28.9)	26 (38.2)	10 (31.3)	0.598+
University, 94 (64.8)	32 (71.1)	41 (60.3)	21 (65.6)	

**Comorbidities 14 (9.7)**	5 (11.1)	7 (10.3)	2 (6.3)	0.871+

**Polypharmacy, 8 (5.5)**	3 (6.7)	4 (5.9)	1 (3.1)	0.900+

**Antiacne medications, n (%)**				

Oral isotretinoin, 77 (53.1)	20 (44.4)	35 (51.5)	22 (68.8)	0.102^

Topical retinoid**, 56 (38.6)	22 (48.9)^a^	27 (39.7)a,b	6 (18.8)^b^	***0.025*** ^

Topical antibiotic**, 32 (22)	16 (35.6)^a^	14 (20.6)a,b	2 (6.3)^b^	***0.009*** ^

BPO/clindamycin**, 30 (20.7)	16 (35.6)^a^	12 (17.6)a,b	2 (6.3)^b^	***0.005*** ^

Oral doxycycline**, 30 (20.7)	2 (4.4)^a^	20 (29.4)^b^	8 (25)^b^	***0.005*** ^

Topical ab/Topical retinoid/BPO, 28 (19)	15 (33.3)^a^	11 (16.2)^b^	2 (6.3)^b^	***0.008*** ^

Oral ab/Topical retinoid, 18 (12)	1 (2.2)^a^	13 (19.1)^b^	4 (12.5)a,b	***0.018*** +

Topical erythromycin/tretinoin, 7 (4.8)	2 (4.4)	5 (7.4)	0	0.276+

**Guidelines**				

ADA 2016** (general concordance)	33 (73.3)^a^	35 (51.5)^b^	22 (68.8)a,b	***0.043*** ^

ADA 2024** (general concordance)	42 (93.3)^a^	58 (85.3)a,b	22 (68.8)^b^	***0.014*** ^

NICE** (general concordance)	12 (26.7) a	24 (35.3) a	22 (68.8) b	***0.001*** ^

TDA** (general concordance)	17 (37.8) a	25 (36.8) a	26 (81.3) b	***<0.001*** ^

ADA** (oral ab concordance)	0a,b	11 (55)^b^	0^a^	***0.009*** +

NICE** (oral ab concordance)	0a,b	11 (55)^b^	0^a^	***0.009*** +

TDA (oral ab concordance)	0	13 (65)	4 (50)	0.236+

ADA 2016** (oral retinoid concordance)	11 (55)^a^	12 (34.3)^a^	22 (100)^b^	***<0.001*** +

ADA 2024 (oral retinoid concordance)	20 (100)	35 (100)	22 (100)	NA

NICE** (oral retinoid concordance)	11 (55)^a^	12 (34.3)^a^	22 (100)^b^	***<0.001*** +

TDA** (oral retinoid) concordance)	11 (55)^a^	12 (34.3)^a^	22 (100)^b^	***<0.001*** +

ADA** (topical ab concordance)	16 (100)^a^	12 (85.7)^a^	0^b^	***0.002*** +

NICE (topical ab concordance)	1 (6.3)	1 (7.1)	0	1+

TDA (topical ab concordance)	0	0	0	NA

ADA** (topical retinoid concordance)	21 (95.5)^a^	22 (81.5)^a^	0^b^	***0.001*** +

NICE (topical retinoid concordance)	0^a^	11 (40.7)^b^	0a,b	***0.001*** +

TDA (topical retinoid concordance)	6 (27.3)	13 (48.1)	4 (66.7)	0.162+

ADA** (BPO concordance)	16 (100)^a^	12 (100)^a^	0^b^	***0.002*** +

NICE (BPO concordance)	1 (6.3)	1 (8.3)	0	1+

TDA (BPO concordance)	0	0	0	NA

* Kruskal–Wallis test

^ Pearson’s chi-squared test

+ Fisher’s exact test

** Different superscript letters indicate statistically significant pairwise differences between groups after Bonferroni-adjusted post hoc comparisons; values sharing the same letter are not significantly different.

ab Antibiotic, BPO: Benzoyl peroxide, ADA: American Dermatology Association, NICE: National Institute of Health and Clinical Excellence, TDA: Türkiye Dermatology Association, IQR: Interquartile range. Bold and italic values indicate statistically significant results (p < 0.05)

**Table 2:** Association of antiacne medications with age and sex.

Drugs	Median age of users (IQR)	p*	Male among users n (%)	Female among users n (%)	p^
Doxycycline	19.5 (17–22)	0.650	11 (35.5)	19 (16.7)	***0.022***
Topical retinoid	19.5 (18–21)	0.654	15 (48.4)	40 (35.1)	0.176
Benzoyl peroxide/clindamycin	20 (18–20)	0.990	5 (16.1)	25 (21.9)	0.48
Oral isotretinoin	20 (18–21)	0.876	15 (48.4)	62 (54.4)	0.553

* Mann–Whitney U test

^ Pearson’s chi-squared test.

IQR: Interquartile range. Bold and italic values indicate statistically significant results (p < 0.05)

**Table 3:** Association between prescribed antiacne therapy patterns and overall concordance status according to each guideline.

Medication pattern	ADA 2016 concordance n (%)	p	NICE concordance n (%)	p	TDA concordance n (%)	p
**Monotherapy**						
Oral retinoid (n=77)	45 (58.4)	0.338^	45 (58.4)	***<0.001*** ^	45 (58.4)	***0.003*** ^
Oral antibiotic (n=10)	0 (0)	***<0.001*** *	0 (0)	***0.006*** *	0 (0)	***0.002*** *
Topical retinoid (n=7)	6 (85.7)	0.253*	0 (0)	***0.042*** *	6 (85.7)	0.051*
**Dual therapy**						
Oral antibiotic + topical retinoid (n=18)	11 (61.1)	0.929^	11 (61.1)	0.051^	17 (94.4)	***<0.001*** ^
Topical antibiotic + BPO (n=2)	2 (100)	0.526*	2 (100)	0.158*	0 (0)	0.498*
**Triple therapy**						
Topical antibiotic + topical retinoid + BPO (n=28)	26 (92.9)	***<0.001*** ^	0 (0)	***<0.001*** ^	0 (0)	***<0.001*** ^
Oral antibiotic + topical antibiotic + topical retinoid (n=2)	0 (0)	0.142*	0 (0)	0.517*	0 (0)	0.498*

Values indicate the number (%) of patients within each treatment pattern classified as concordant with the respective guideline. p values compare concordant versus nonconcordant prescribing across treatment categories using Pearson’s chi-squared or Fisher’s exact test, as appropriate.

* Fisher’s exact test,

^ Pearson’s chi-squared test.

BPO: Benzoyl peroxide; ADA: American Academy of Dermatology; NICE: National Institute for Health and Care Excellence; TDA: Türkiye Dermatology Association. Bold and italic values indicate statistically significant results (p < 0.05)

**Table 4:** Warnings provided for antiacne treatments and their frequencies conveyed to patients.

Drug & warning item	n	Percentage of counseling
**Doxycyclin; median (IQR), mean±SD**	**30**	**60 (40–60), 49.3±18.7**
Use sunscreen during treatment	27	90
Take in an upright position	23	76.7
Take with plenty of water	21	70
If coadministered with antacids, iron, or bismuth subsalicylate, take 2 h before or 4 h after	3	10
Inform physician/pharmacist if severe diarrhea occurs	0	0
**Oral Isotretinoin; median (IQR), mean±SD**	**77**	**71.4 (71.4–71.4), 72.3±4.8**
Do not become pregnant while taking this medicine	77	100
Have regular blood tests during treatment	77	100
Use sunscreen during treatment	77	100
Do not discontinue treatment (16–24 weeks) without consulting physician	77	100
Consult physician if muscle pain occurs (morning or at rest)	77	100
May cause skin irritation and dryness	76	98.7
Do not donate blood while taking this medicine	4	5.2
Use foods or supplements containing vitamin A with caution	2	2.6
**Benzoyl peroxide/clindamycin; median (IQR), mean±SD**	**30**	**62.5 (62.5–65.6), 62.08±12.05**
Use sunscreen during treatment	30	100
Use at different times if combined with a topical retinoic acid derivative	28	93.3
Continue treatment for at least 4 weeks	28	93.3
May cause skin irritation and dryness	26	86.7
If irritation is severe, apply every other day or less frequently	26	86.7
Avoid contact with mucous membranes, lips, and eyes	7	23.3
May bleach hair or clothes upon contact	3	10
Apply to the entire face	1	3.3
**Topical retinoid; median (IQR), mean±SD**	**55**	**75 (75–87.5), 74.7±14.1**
Apply the medicine in the evening	55	100
Apply to the entire face	54	98.2
May cause skin irritation and dryness	53	96.4
If irritation is severe, apply every other day or less frequently	52	94.5
Use sunscreen during treatment	51	92.7
Continue treatment for at least 12 weeks	40	72.7
Avoid contact with mucous membranes, lips, and eyes	24	43.6
Skin may lighten or darken during treatment	0	0

IQR: Interquartile range, SD: Standard deviation

## Data Availability

Anonymized data are available from the corresponding author upon reasonable request.
